# Steel Automotive Wheel Rims—Data Fusion for the Precise Identification of the Technical Condition and Indication of the Approaching End of Service Life

**DOI:** 10.3390/ma17020475

**Published:** 2024-01-19

**Authors:** Michal Borecki, Arkadiusz Rychlik, Li Zan, Michael L. Korwin-Pawlowski

**Affiliations:** 1 Institute of Microelectronics and Optoelectronics, Warsaw University of Technology, 00-662 Warszawa, Poland; 2 Faculty of Technical Sciences, University of Warmia and Mazury, 10-719 Olsztyn, Poland; 3 Institute of Telecommunications, Warsaw University of Technology, 00-661 Warszawa, Poland; 4 Département d’informatique et d’ingénierie, Université du Québec en Outaouais, Gatineau, QC J8X 3X7, Canada

**Keywords:** steel, wheel rim, technical condition, data fusion, dispersion of damping factors

## Abstract

Steel automotive wheel rims are subject to wear and tear, down to the end of their service life. Manufacturers use standard destructive tests to determine the probable lifetime of the car wheel rim. With this approach, to predict the remaining use time, it is necessary to know the initial parameters of the wheel rim, actual mileage, and its use characteristics, which is difficult information to obtain in the real world. Moreover, this work shows that a vehicle’s technical condition can affect the rim’s remaining service time. This work describes a new method of precise binary identification of the technical condition of steel car wheel rims using the dispersion of damping factors which result from experimental modal analysis. This work also proposes a new method of indicating the approaching end of wheel rim service life with limited parameters: run-out, average of damping factors, and dispersion of damping factors. The proposed procedure requires two sequential examinations of the rim in standard periods related to the average annual mileage of the vehicle. On this basis, it is possible to indicate the approaching end of the life of the steel rims about 10,000 km in advance.

## 1. Introduction

The wheel is an essential part of the vehicle. It can be made as a monolithic structure, such as a train wheel [1], or a hybrid construction, such as an automotive wheel, which consists of a tire and a rim [2]. The automotive wheel rim allows the tire to be fixed to the rim and the wheel to the hub. Automotive wheel rims can be made in different configurations [3]. A simple classification of automotive wheel rims includes single-piece and multi-piece types. The single-piece constructions can be realized as a cast from a mold or machined from solid material. The standard design of the multi-piece wheel rim includes the use of different materials, such as various types of steels, aluminum and magnesium alloys, and glass or carbon fiber components used for metal reinforcement [4]. As the density of steel is almost 2.5 times that of aluminum, automotive wheel rims made of these materials behave differently in use.

**Figure 1:**
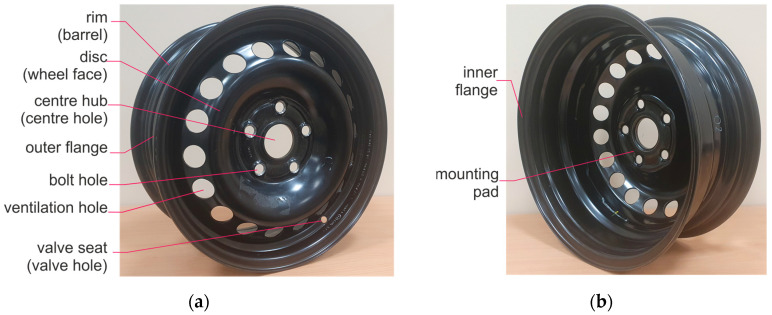
View of a steel automotive wheel rim: (**a**) Outer view; (**b**) Inner view.

A view of a steel automotive wheel rim with a glossary is presented in Figure 1. The wheel rim is not an ideal axis-symmetric structure. Axial symmetry is disturbed by the ventilation holes, bolt holes, and valve seat. The number of bolt holes and their shape can differ depending on the automotive manufacturer. The bolt hole can be conically or cylindrically shaped.

Due to the complex geometry of the structure, the investigation and optimization of automotive wheel rims’ construction are currently conducted by computer-aided simulations using finite element method (FEM). There are two basic types of FEM used in wheel rim simulations that result in a determination of responses for forced vibrations and loads.

The vibration analysis results obtained from FEM are displacement, velocity, and acceleration of nodes in a time function. Vibration data can be analyzed in the frequency or the time domain. In contrast, modal analysis is a process of obtaining modal parameters such as natural frequencies, damping loss factors, and modal constants from the vibration data. Natural frequencies are mainly related to object shape, dimensions, and material [5]. The modes of complex geometrical objects are numbered according to the increasing resonant frequency of vibrations [6].

The damping factor of a mechanical object depends on three main effects: the internal friction of the material, the acoustic radiation of the system, and the energy loss caused by boundary connections of the system [7]. Generally, the component of internal friction of material dominates over other factors [8]. In the macroscopic scale, the damping loss factor increases when Young’s modulus decreases. However, when the object is not symmetrical, the damping factor is characterized by a set of values [9]. Thus, in a precise analysis of complex structures, the damping factors are not always characterized by a monotonic course [10]. The 30% damping factor dispersion and some dispersion of the natural frequency can be measured in specified short-term situations of mechanical objects. However, in standard cases, the damping loss factor increases with the accumulated fatigue of the system and knowing the start and end values, the trajectory of the damping factor can be used to predict the time to failure of mechanical objects [5].

The mechanically forced fatigue of an object can be followed by self-healing when the object is not operated. The short-time healing state of the mechanical properties of the object may be associated with the presence of local maxima of the damping factor that are followed by an increase in the resonant frequency [11]. The long-term result of the healing of mechanical objects is commonly related to a decrease in damping factor and stabilization of natural frequency. While the precise interpretation of the experimental results of the modal analysis is not trivial, a system’s damping factors depict the mechanical object’s structural integrity, and modal analysis can be essential to characterize the wheel rim’s actual technical condition.

The loading analysis of automotive wheel rims performed with FEM enables the calculation of forced total deformation, equivalent stress, and equivalent strain [12]. Obtained mechanical properties are fundamental for correctly assessing the lifespan of the wheel rim for the loaded car in a stable position and in motion [13]. After simulations, automotive wheel rim investigation includes performing some destructive tests [14]. For example, experimental axial or radial axle load of the rim is performed using dedicated equipment that consists of a controlled actuator and a deformation measuring system [15]. The standard investigation of the wheel rim involves a rolling test, cornering test, and radial fatigue test [16]. Deformations obtained from the tests can be recorded via an optical 3D measuring system [17].

In all constructions, the technical condition of a component relates to its initial state and wear state [18]. Producers may examine the initial state of the steel automotive wheel rim using measurement equipment for the dimensional control of rim and disc, unbalanced measurement, air tightness test oriented to search for cracks, and the rolling test for pointing resistance for radial fatigue [19]. During regular operation, the wheel rim is exposed to standard wear related to its operation conditions, periodic tire changes, wheel balancing, accidental direct impacts, and impacts transmitted by the tire.

The degradation of the automotive wheel rim in natural conditions is correlated with the load and the vehicle’s speed and type of route, whether a highway or an urban artery. Monitoring the vehicle’s course in the city is not trivial [20]. Camera observation allows for the estimation of vehicle acceleration and lane-changing dynamics only at the place of observation [21]. Approximate results indicate that cars move on motorways mainly on almost straight sections, and the change in direction is associated with lane changes and lasts about a few seconds, while the average number of lane changes is up to a dozen or so per hour [22,23]. The ratio of time of vehicle motion on a curve to linear motion can be estimated by analyzing a typical one-hour drive, where a quick lane change takes about 4 s. On the motorways, the driver swiftly changes lanes about ten times per hour. Thus, the ratio can be estimated for motorways as 0.01. The number of quick lane changes in the city is around 100 times per hour. Therefore, the ratio should be increased to 0.1. Therefore, the average curvature to linear movement ratio can be estimated as 0.055. According to the physics of circular motion, higher forces act on automotive rims in a quick lane change than in a slow change. Hence, simulators of accelerated rim wear use forced wheel movement along a circular trajectory or the radial fatigue test to simulate the dynamics of the wheel during cornering [24]. The expected results of these tests are the internal stress of the rim [25].

Internal stress can lead to the first visual sign of wheel rim wearing: finish layer peeling [26]. On the other hand, when the finish layer of the wheel rim is cracked, the metal construction begins to erode in some road conditions with a speed of 0.1 mm/year [27]. Loss of material due to corrosion is an issue in the area where the wheel rim itself mates to the hub and under the nuts securing the wheel to the hub [28,29]. Standard procedures of wheel care, including regular tire change and wheel balancing, require wheel removal from the hub and wheel mounting on the hub [30]. This process requires tightening wheel locking nuts with a defined torque, which can result in degrading the areas under the nuts and sides of corresponding holes [31]. The result can be the plastic deformation of bolts, nuts, and the area under them [32], and further missing nuts, which can have severe implications [33].

Accidental situations of wheel rim degradation most often occur due to hitting straight-on potholes, curbs, or other obstacles on the road, or side collisions with the curb [34]. Possible results are visible and internal cracks in the metal, bent inner or outer flanges, and deep marks on the surface [35]. In most cases, the leading cause of severe damage is driver negligence and underinflated tires [36]. However, when the wheel rim is worn and overloaded, the probability of a critical break due to an impact increases [37].

The last stage of rim degradation is its complete uselessness or destruction. Destruction of the wheel rim can be defined as visible signs of deformation and damage to the material structure [38]. Thus, the binary classification of rim technical condition as fit to use or damaged was proposed. The technical condition of the automotive rim can be examined using non-destructive vibration analysis performed in laboratory conditions [39]. Suggestions to improve the analysis include modifying the measuring system using a wheel balancer to a rim condition measurement system that supports run-out and vibration modes at four rim points and recording them in a dedicated database. These data are subject to further processing with an artificial neural network (ANN) [40]. In the indicated paper, the ANN was trained with a set of data obtained for new and used rims. The raw vibration data were obtained for vibration excitation at four points on each rim. The raw data were reduced to pattern form for the ANN training process and implementation task. Different types of data patterns were investigated. The analysis shows that automotive wheel rim fit for use can be characterized by a set of actual and reference parameters of experimental modal analysis, including the dispersion of excited natural frequencies and their amplitudes. However, a significant disadvantage of this system was the requirement of absolute values of amplitudes of vibration measurements at an exact time. Therefore, a precise mechanism of vibration excitation, its calibration, and synchronization with the data acquisition system were important.

The field tests of automotive rims are possible only for complete wheels and require constructing an attached device whose main component is a dedicated vibration-sensing device. The examination results show that the frequency spectrum of wheel fit for use is below 5 Hz. At the same time, precise results indicate that the vibration signal is expected between 0 and 1.65 Hz with a main peak around 0.1 Hz [41]. A similar set-up based on an integrated acceleration measurement system and ANN signal processing may be used to detect the bolts’ loosening securing the rim to the hub [42].

The long-term wear of rims is today mainly analyzed for wheels of high-speed trains [43]. The analysis shows that the dominant fatigue process of steel train wheel rims is micro-damage evolution under off-axis fatigue [44]. In the study of the prediction of railway wheel service life, difficulties related to the availability of samples with a known type of degradation and wear state were indicated, as well as the necessity of the precise definition of their wear examination intervals [45]. It has also been shown that the prediction of the remaining useful life of the railway wheel, based on small samples, leads to significant discrepancies in theoretical and real results [46]. Based on the above premises, it has been proven that generating examples of railway wheels that comply with the standard degradation distribution is an essential step in predicting the lifetime of any railway wheel [47]. So far, deterministic procedures for predicting a railway or automotive wheel’s remaining life are unknown.

In most cases, car owners and vehicle inspection station employees determine an automotive wheel’s technical condition based on its visual inspection and the soapy water test oriented to the detection of air leaks [48]. The check should also concern the inside section of the rim and the sides that are in contact with a dual wheel case [49]. Sometimes, visual inspection is followed by examining the wheel rim geometry, a standard approach to assessing the technical condition of an automotive wheel rim. Axial and radial run-out values of the rim on the inner and outer sides of the rim flange relative to the centering hole must be lower than {2.0 mm; 1.2 mm} according to the ETRTO standard [50]. Other standards exist as country regulations, such as Poland’s PN-93S standard {1.25 mm; 1.25 mm} [51]. Excessive outer rim flange deformations with run-out values greater than 2.2 mm can be felt as vehicle user’s steering wheel vibrations [13]. The wear examination intervals relate to seasonal tire replacements or annual vehicle technical inspections. It can be concluded that even though the current recommendations vary, and the test methods are not consistent, wheel owners need non-destructive testing to indicate the technical condition and remaining life of the automotive wheel rim or at least to point to the moment in time when the rim must be changed [52].

## 2. Methodology of Examinations and Following the Structure of the Publication

The classic methodology of wheel rim examination used by manufacturers consists of destructive tests, e.g., ISO 3006 [53], SAE J267 [54], or SAE J328 standards [19], to determine the lifetime of the produced series. Thus, the maximum expected distance to drive in standard conditions is known. However, the situation on the roads is not really known and the actual technical condition of the rim is not a linear function of distance traveled. To predict the remaining distance of use of the rim, the distance that was traveled and the characteristics of previous use of the wheel rim must be known. The characteristics of prior use are often unknown. Thus, much information in automobile blogs points to the lifetime of car wheel rims from 4 years up towards almost infinity [55]. Since steel is the most common material used in automotive rims, the proposed study is limited to rims made of this material. A rim made of aluminum–magnesium alloy has a longer lifetime than a steel rim [56].

In this work, a comparison of the results of destructive tests with non-destructive measurements of steel car wheel rims is provided. The investigation considered cyclic destructive fatigue tests separated by non-destructive classical visual inspection, rim run-out measurements, and the application of experimental modal analysis. The presented examinations were limited to the widely used sizes of steel rims in passenger cars and trolleys of two diameters: 14 and 15 inches. The automobile industry does not define matching rules between wheel hub construction and car types [57]. Therefore, 15-inch steel automotive rims of type 6Jx15H2 used in popular cars and 14-inch rims of type 51/2Jx14H2 often mounted in small vehicles and trailers were investigated. The rim type 6Jx15H2 can be described as a 15-inch rim with a 6-inch-wide barrel, see Figure 1.

The selection of rims for examination was limited to the so-called “second assembly” type. This was due to the regulations that mandatory tests apply only to wheel rims marked as original that are mounted and sold with a new vehicle [58]. New automobile wheel rims commercially available at the original equipment manufacturer, despite showing the same markings, often differ from those that are mounted on vehicles at the factory. Rims under investigation were second assembly type and were made by an original equipment manufacturer (OEM), an acceptable replacement manufacturer (ARM), or a low-cost manufacturer (LCM). The examinations of steel automotive rims of new or used conditions with the characterized initial conditions according to standards are presented in Table 1.

The run-out examinations were performed using eddy current sensors type CW10 coupled to NI card type USB 6343, which was part of the measurement system for experimental modal analysis. The initial run-out examination showed that the rims of the second assembly met the European ETRTO standards when examined in factory conditions. However, parameters of rims from ARM sometimes did not meet the standards but the run-out values on any flanges were below 2 mm. In contrast, the rim of the LCM’s initial parameters could surprise both positively and negatively.

These rims were examined with experimental modal analysis (EMA) and run-out methods, while fatigue tests of the rims were conducted in laboratory or as on-field tests. The methodology of examination corresponded to the sections of this publication as follows:

- The initial numerical modal analysis of the rims necessary for defining the acquisition system parameters used for EMA is presented in Section 3;
- The construction of a measurement system for EMA based on an exciter with repeatable parameters and assumed precision of acquisition data is presented in Section 4;
- The construction of a fatigue station aimed at performing an adapted cornering fatigue test is depicted in Section 5;
- A series of examinations of rims with the use of a fatigue station and EMA set-up based on step-by-step sequential fatigue procedure with initial and sequential rim state characterization with EMA and run-out methods are depicted in Section 6;
- The discussion of laboratory test results and on-field testing are presented in Section 7;
- The conclusions are given in Section 8.

## 3. Initial Numerical Modal Analysis—Materials and Constructions

The modal analysis was selected to determine the initial investigations. However, the use of a primary function that describes normal modes of automotive rim components, coupled with linear vibration theory and linear superposition, seems to not be recommended [59]. Therefore, the SOLIDWORKS software (Simulation Premium 5.0 or SP5.0) has been used to provide a comprehensive set of structural and finite element analyses and to analyze the natural frequency of the object, which was modeled using a solid mesh. Five eigenfrequencies were searched using the FFEPlus solver (Intel Sparse, https://help.solidworks.com/2019/english/SolidWorks/cworks/c_Analysis_Solvers.htm, (accessed on 14 November 2023).

Numerical mode analysis was conducted for rim type 6Jx15H2; see Figure 1. A solid mesh generated for mixed curvature was used to model the rim geometry. The maximum mesh element size of 1.76098 mm and the minimum element size of 0.352197 mm were indicated. The mesh generator created a mesh of 1,882,950 elements and 3,082,794 nodes. The maximum aspect ratio of an element was 9.6918. Carbon steel (SS) type 1023 sheet was assumed as the rim model material. The structure type of the model was defined as linear elastic and isotropic. The material parameters were given as: specific mass: 7.858 kg/m^3^, yield strength: 2.82685 × 10^8^ N/m^2^, tensile strength: 4.25 × 10^8^ N/m^2^, longitudinal elasticity coefficient: 2.05 × 10^11^ N/m^2^, Poisson’s ratio: 0.29. In the simulation model, the stationary area was the hub contact area.

**Figure 2:**
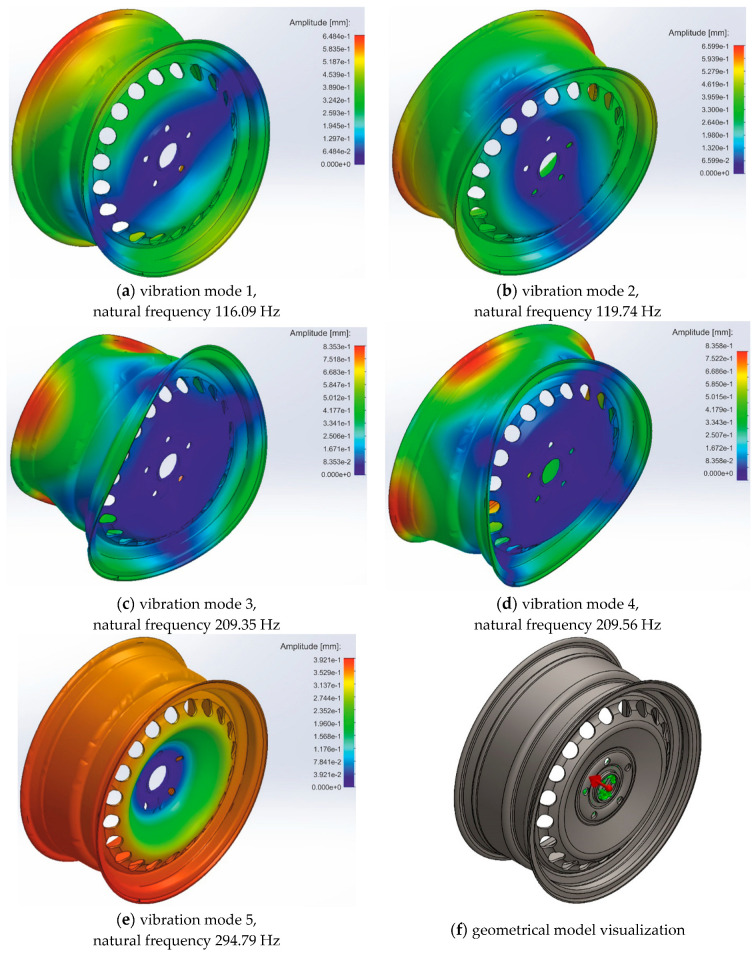
Set of simulation results in natural frequencies of the rim in the ideal shape: (**a**) Vibration mode 1; (**b**) Vibration mode 2; (**c**) Vibration mode 3; (**d**) Vibration mode 4; (**e**) Vibration mode 5; (**f**) Geometrical model visualization.

The results of the simulation of the ideal-shape rim are presented in Figure 2. The simulation analysis of the excited modes indicates that the fundamental modes occur in pairs having slightly different resonance frequencies. The mentioned difference for modes 1 and 2 is 0.65 Hz, and for modes 3 and 4, it is 0.21 Hz. Notably, greater amplitudes of vibration characterize modes 3 and 4 more than modes 1 and 2. Therefore, modes 3 and 4 are more likely to be excited than 1 and 2. The lowest amplitude of vibration characterizes mode 5. Similar modes can produce differences and sum frequency components in use. It is essential because the results of frequency difference are always at the low-frequency band.

**Figure 3:**
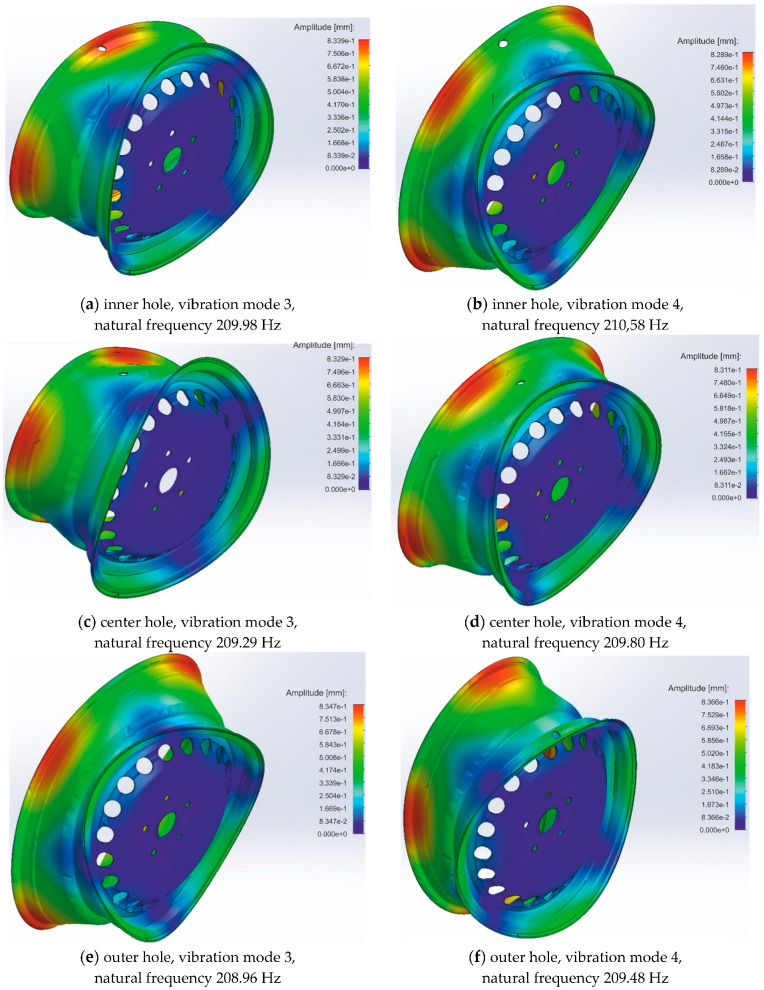
Simulation results of modes 3 and 4 of the rim-degraded situation with the hole positioned in the barrels: (**a**) Hole in inner section, mode 3; (**b**) Hole in inner section, mode 4; (**c**) Hole in center section, mode 3; (**d**) Hole in center section, mode 4; (**e**) Hole in outer section, mode 3; (**f**) Hole in outer section, mode 4.

The intentionally degraded rim was simulated to show the low bandwidth of the EMA. The degradation was a 15 mm diameter hole in the rim barrel positioned in the barrel’s inner, center, and outer sections. Selected results of the simulation of modes 3 and 4 are presented in Figure 3. The obtained results show that the natural frequency of modes 3 and 4 is similar in all cases, but when comparing Figure 2 and Figure 3, the values of natural frequencies difference between modes are more remarkable in the rim-degraded case than in the ideal case.

**Figure 4:**
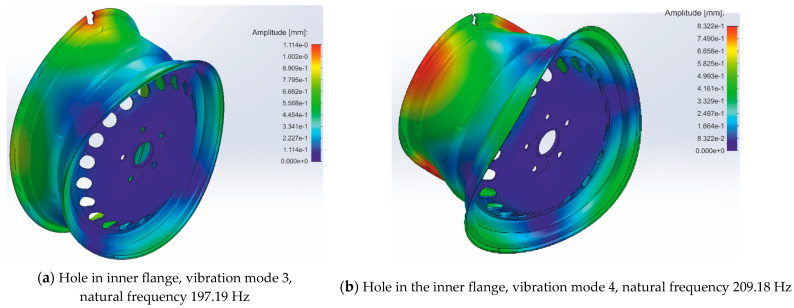
Simulation results of a severely damaged wheel rim: (**a**) Hole in inner flange, vibration mode 3; (**b**) Hole in the inner flange, vibration mode 4.

A severely damaged rim can also be described as a rim with a broken-out part of the inner flange. This fact can be simulated with the use of two holes with a diameter equal to 15 mm, as assumed in the previous analysis. Such damage is easily overlooked during inspections. In addition, due to its size, such damage leads to air escaping from the tire. The simulation results of a rim with such damage are shown in Figure 4.

The summary of the obtained results is presented in Table 2. As can be seen, the direct analysis of the amplitudes and frequencies of the excited modes for individual cases does not show regular dependency damage to amplitude tendency. On the other hand, studying the variability of the maximum amplitudes and frequencies of the excited modes indicates the possibility of detecting rim damage. As the frequency difference signal is cast in a low band, the presence of a signal in the low band can be used as an indicator of rim damage. Thus, the experimental modal analysis set-up should be characterized by the possibility of measurement in the low band of frequency signals in the range of from 0 to 12 Hz with at least 0.1 Hz resolution of frequency examination. According to the simulation results (see Table 2), measuring modal parameters as the variability of maximum amplitudes is much more complex than the variability of natural frequencies of excited modes.

## 4. Measurement System for Experimental Modal Analysis (EMA) and Run-Out of Rim

**Figure 5:**
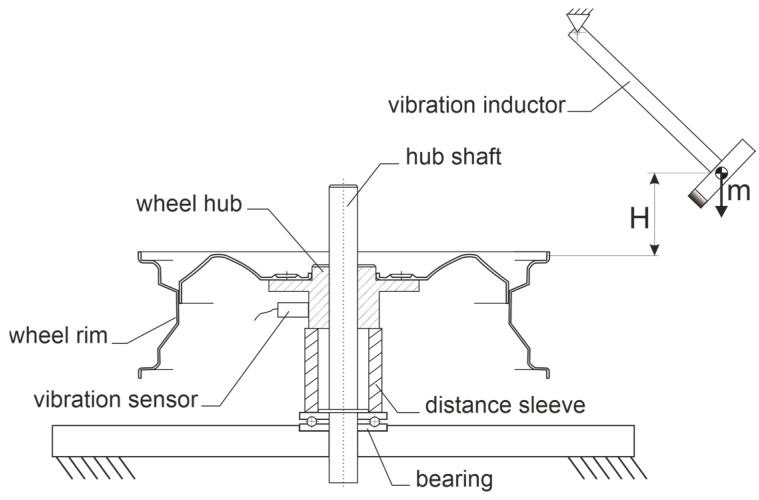
Scheme of the mechanical components of the measurement system used for experimental modal analysis.

**Figure 6:**
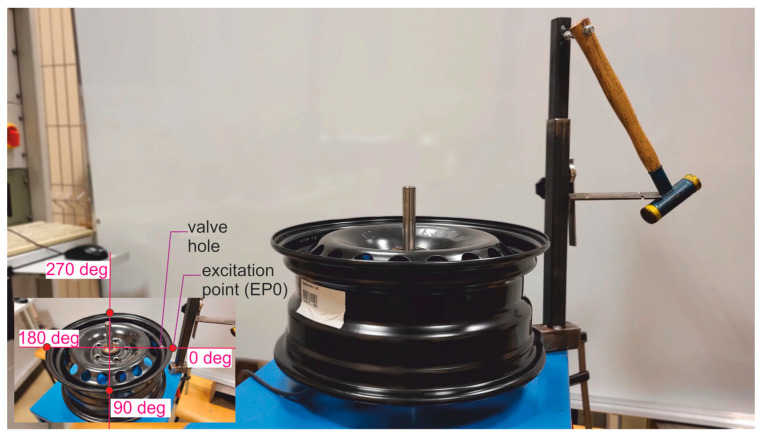
View of the mechanical part of the measurement system for EMA with vibration excitation points (EP) marked.

The measurement of experimental modal analysis based on monitored vibration excitation of the rim is presented in Figure 5. The view of the mechanical part of the measurement system for EMA is presented in Figure 6.

The steel automotive wheel rim is positioned on the hub shaft and fixed by gravity on the hub. The vibration inductor is a roofing hammer of a mass of 425 g that is lifted before the excitation of vibrations to the height of H = 26 cm. In such conditions, the impact energy is 1.08 J.

The vibration sensor consists of the ICP accelerometer head model 625B01 attached to the profiled magnetic bed with a mounting screw. The head is characterized by a ±50 g measurement range, 100 mV/g sensitivity, and linear (±3 dB) frequency measurement range from 0.2 to 10,500 Hz. The interface between the head and computer is the NI USB 6343 acquisition card from National Instruments. Due to the limitation of the linear bandwidth for low frequencies, a calibration curve for the sensor was developed for operation in the range of 0.02–10 Hz. Acquired signals of amplitude versus time are used to determine logarithmic decrements of damped oscillations as well as to calculate frequency responses with the use of Fast Fourier Transformation (FFT). The logarithmic decrements are used further to calculate the damping factors. These calculations are performed on a personal computer in the LabView software (Version 2021).

Shaft rotation was measured with an encoder with a resolution of 1.40°. Vibrations were induced sequentially for four excitation points, defined by angles of wheel shaft rotation of 0, 90, 180, and 270 degrees. The 0-degree position was determined by the valve hole located on the excitation axis.

**Figure 7:**
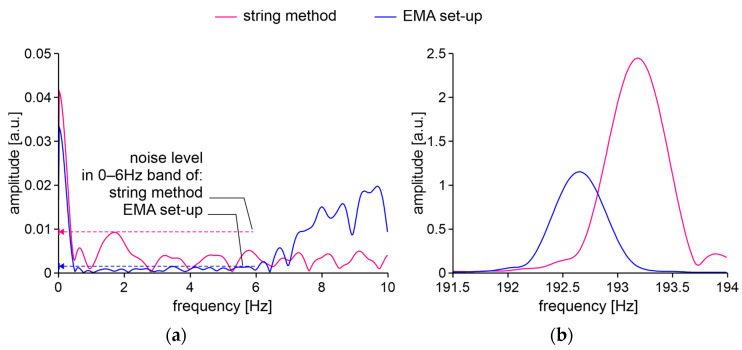
Ideal OEM rim examination with EMA set-up and using the string method: (**a**) Low band; (**b**) Natural frequency band.

Example results of spectra investigation with the resolution of 0.02 Hz, using the proposed set-up and the string method for an ideal rim, are presented in Figure 7. The string method was called here the measurement of vibrations of a hanging rim, which was positioned on the hub and shaft to which the string was attached. The mentioned string was attached to the ceiling on the other side. The expected mathematical model maximum value does not characterize the constant component (at frequency 0 Hz) due to head coupling with the use of a capacitor to the acquisition card. In the analyzed case, the dominant frequency peak measured in the EMA set-up is 192.60 Hz, and for the string method is 193.3 Hz. The difference is most likely due to the influence of the bearing, which was not present on the shaft in the string method. Additionally, both values are lower than the initial simulations. The differences in natural frequencies of simulation and measurement come from the measurement of the wheel rim coupled with mounting accessories and simulation of only the wheel rim.

In the 0–6 Hz band, the noise level for the string method is 4.5 times greater than for the one obtained from the EMA set-up. Thus, the EMA set-up was used for examinations of typical degraded automotive wheel rims. Moreover, based on measurement characteristics obtained with the acquisition system, for the mentioned vibration excitation points, the damping ratios on natural frequencies are calculated using logarithmic decrement of amplitudes [60].

The results obtained in ten series of measurements of the natural frequency and damping factor sets for four excitation points of the wheel rim from OEM, which is in factory condition, are similar but not identical. The excited frequencies differ maximally by 0.09 Hz. Thus, the low band result of such frequencies mixing can be masked in the measurement set-up by the actual constant component of Fourier transformation. Relating this difference to the resonant frequency gives a relative dispersion of 0.05%. In contrast, the dispersion of the damping factor is 11.4%.

Therefore, in the proposed EMA set-up, besides the average values of natural frequencies (*aNF*) and the average of damping factors (*aDF*) for the set of excitation points, the difference in natural frequencies (Δ*FN*) and dispersion of damping factors (*dDF*), and presence of a signal in the low band are results of measurements. The difference in natural frequencies is calculated according to the equation:

(1) $$\Delta FN=\max\left(FN\right)-\min\left(FN\right),$$

where *FN* is the set of natural frequencies obtained for the set of excitation points (EP), while max(x) and min(x) are standard mathematical functions. The dispersion of damping factors is calculated with the equation:

(2) $$dDF=\frac{\max\left(DF\right)-\min\left(DF\right)}{\mathrm{avg}\left(DF\right)},$$

where *DF* is the set of damping factors obtained for the set of excitation points (EP), and avg(x) is the standard mathematical function for calculating the average value of the set.

## 5. Fatigue Station

The dynamic wheel cornering fatigue test is one of the most significant tests to determine the usability expectation for newly manufactured automotive wheel rims. This test can be implemented with a rotating table configuration or as the rotating bending test [61]. The rotating bending test allows for the evaluation of the resistance of the automotive wheel rim to a bending moment achieved via an arm equipped with a rotating unbalanced mass. This mass is called eccentric or dead mass and is mounted on the load cell [62]. A strain developed at the rim due to the moment applied at the mounting pad may differ from case to case as 13%, while the moment applied to the mounting pad and disc results in a strain difference of 2% [63]. Thus, for precise examinations, the moment should be applied at the mounting pad and disc [64].

**Figure 8:**
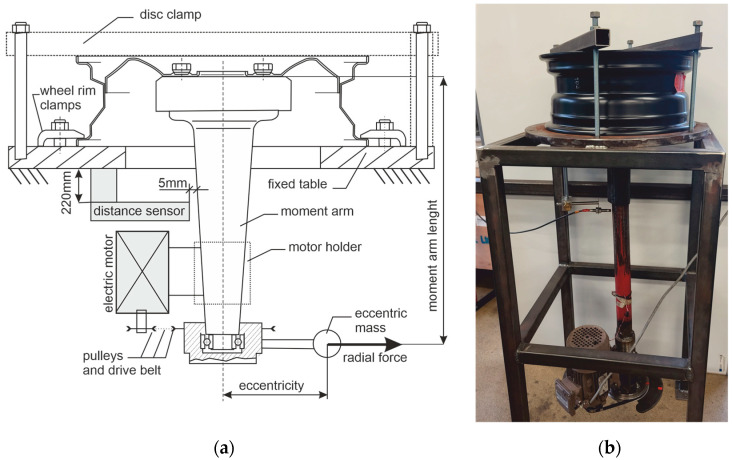
Mechanical functions of the fatigue station: (**a**) Schematic drawing; (**b**) View.

In our examination, we do not intend to follow standards of test implementation directly. The aim of the fatigue station we have built is to perform the accelerated fatigue of steel automotive wheel rims in conditions like the natural exploitation of standard cars. As a standard car, we assume 1500 kg of mass on four wheels based on 15-inch rims. We take that standard turns are made on a circle with a radius of 20 m, with a linear speed of 20 km/h, and the wheels do not skid. For such assumptions and basic estimations, the bending torque acting on the rim is 214 Nm. We also assume that the fatigue station should provide 60 percent of the designated load and run continuously. The proposed value is between what the UN ECE Regulation R124 recommends for replacement wheels, Annex 6, where two independent examinations at 50% and 75% of maximum side force were defined. The fatigue station simplified scheme and its view are presented in Figure 8. During the experiments, the fatigue station was attached to a massive base that weighed 5 tons. The fatigue station of the wheel rim is characterized by the following parameters: moment arm length of 110 cm, eccentric mass of 3 kg, eccentricity of 25 cm, and rotation speed of 450 revolutions per minute. Thus, the bending torque is 129 Nm, which is 60% of the assumed standard cornering of a typical car.

Based on the speed of the standard vehicle movement, a relationship between the duration of the fatigue test and the expected mileage of the vehicle can be estimated. The expected mileage for the test of the assumed vehicle, for a 15-inch wheel rim equipped with a standard tire, 0.055 the average ratio of curvature to linear movement, 450 revolutions per minute, is 1200 km per hour of test. Most used rim testing can be completed twice a year, during tire changes for heavily used vehicles and at the annual examination when all-season tires are in use. Heavy car use during the year in Germany equates to approximately 19,000 km, while the average annual mileage of cars in the European Union can be estimated as 11,000 km [65], where all-season tires are in use. This timestamp of rim examination can be related to 9500 km up to 11,000 km. In our case, we assumed that 8 h of duration of the fatigue test corresponded to 9600 km of expected mileage in mixed traffic conditions.

The fatigue station was equipped with an eddy current distance sensor model CW10 cooperating via the OP200 interface with the input of an acquisition card NI USB 6343 used by the EMA measurement system. A distance sensor was used to monitor the deflection of the torque arm with 2% accuracy. This distance during the rotation of the eccentric mass is correlated with the deflection of the rim disc. In turn, this deflection is related to the material parameters of the wheel. According to the ISO 3006 standard, the rim disc deflection must not increase by more than 20% during the 50,000 eccentric mass rotations test. Exceeding this condition with properly tightened bolts means exceeding the permissible fatigue of the material and is the signal for fatigue procedure termination. Thus, in the fatigue test, it is also essential to maintain the pressure of the bolts. The bolts must be pre-tightened to a torque of 120 Nm and the torque of the screw during the experiment should be between 96 Nm and 120 Nm. The range was tested after 10,000 rotations of eccentric mass, 22 min after the start of the first step of the fatigue procedure. For this purpose, two Jonnesway type T27340N torque wrenches calibrated in accordance with the PN-ISO 6789 standard [66] were used. The first torque wrench was set to 120 Nm, while the second to 96 Nm. The second wrench was used to test the lowest value of the moment. In all the recorded experiments, that value was not crossed. Crossing this value automatically ended the fatigue experiment. However, sometimes the bolts became acceptably loose and had to be tightened with the first wrench with a torque of 120 Nm.

## 6. Experimental Laboratory Results

**Figure 9:**
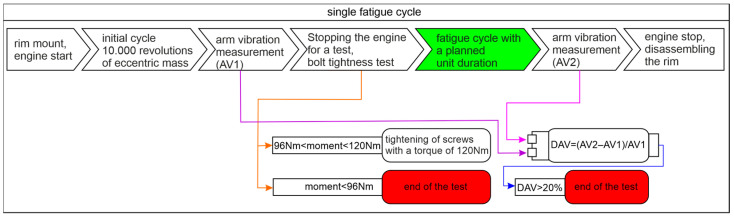
The scheme of a single fatigue cycle.

This section presents detailed data of laboratory examinations based on a single fatigue cycle shown in Figure 9. The fatigue planned unit duration is related to the expected mileage in mixed traffic conditions. The complete cycle of laboratory experimental investigations includes a series of tests consisting of EMA, run-out measurements, and a single fatigue cycle. The process of laboratory investigations continues until one of the end conditions of the test is met.

### 6.1. New Rim Type 6Jx15H2 from OEM Examinations with the Sequential Use of the Fatigue Station

**Figure 10:**
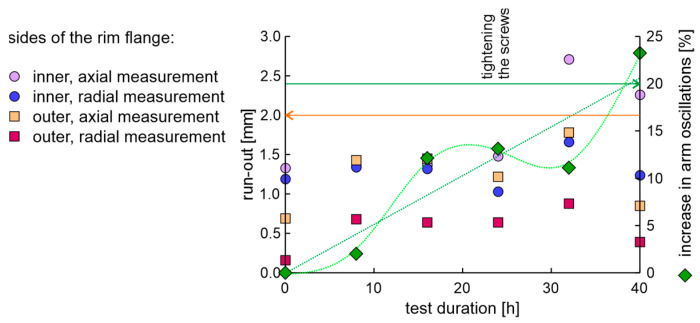
The axial and radial run-out values and increase in moment arm oscillations of the new rim from OEM in a fatigue test.

The rim in the OEM new factory condition was examined using the fatigue station and the EMA and run-out measurement system. The duration of a single fatigue cycle was 8 h. The axial and radial run-out values of the rim on the inner and outer sides of the flange are presented in Figure 10. The condition for the end of the test was met in 40 h, as the increase in moment arm oscillations increased above 20%. The obtained results of run-out values show that after 32 h of testing, the rim lost tolerance of dimensions.

**Figure 11:**
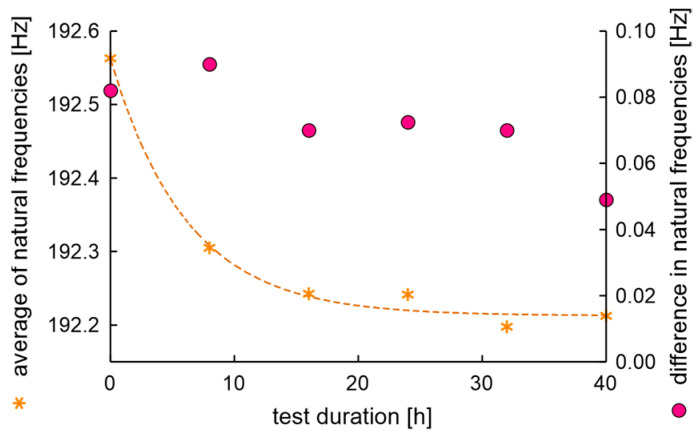
Average values and differences in natural frequencies of the rim of OEM in a standard fatigue test.

The average values and differences in natural frequencies are presented in Figure 11.

**Figure 12:**
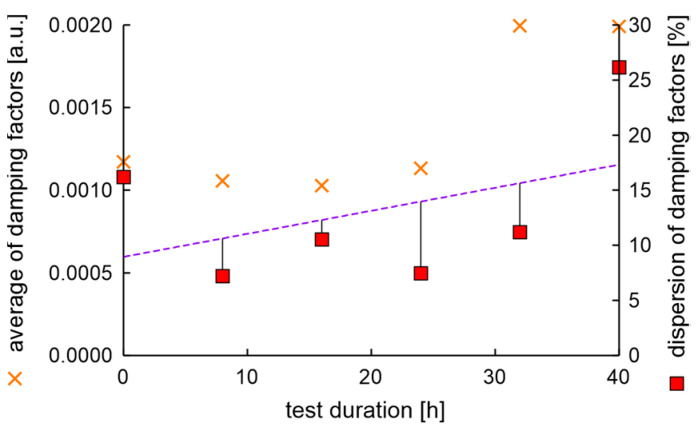
Average values and dispersion of damping factors of the rim from OEM in a standard fatigue test.

In the experiment, the average value of natural frequencies decreased, while the difference in natural frequencies can be characterized by decreased tendency. It can be postulated that the fatigue station performs a peculiar mechanical process related to rim running-in. The average values and dispersion of damping factors are presented in Figure 12.

Results show that the average damping factor increases when the rim loses tolerance of dimensions. In this case, the dispersion of damping factors increases, but with a delay. It can also be seen that the local minimum dispersion of damping factors coincides with the cycle in which the rim mounting bolts were tightened. However, it should be noted that a significant increase in the dispersion of damping factors coincides with an increase in moment arm oscillations above 20%.

### 6.2. New Rim Type 6Jx15H2 from ARM—Sequential Tests Followed by Continuous Tests until the End of Fatigue Test Condition Is Met

**Figure 13:**
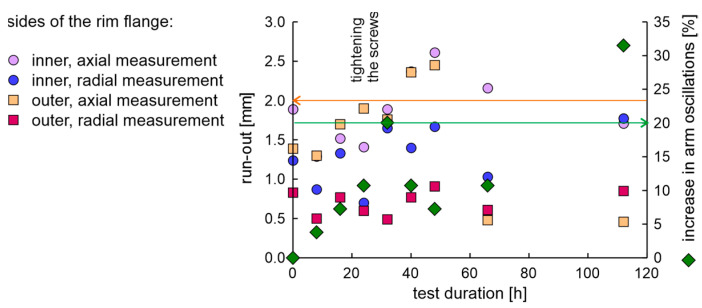
The axial and radial run-out values and increase in moment arm oscillations of the new rim from ARM.

The rim from ARM was examined in a factory-new condition. The axial and radial run-out values of the rim on the inner and outer sides of the rim flange and the increase in moment arm oscillations are presented in Figure 13. The same situation of tightening the screws at the third fatigue cycle occurs for the new rims from OEM and ARM.

Since the increase in moment arm oscillation after 32 h of fatigue is 20% and is not greater than the acceptable moment arm vibration, successive fatigue cycles were performed. The rim run-out that exceeds 2.5 mm occurs at 40 h of fatigue, while the highest values of shape distortion occur for 48 h of test. The time of these distortions is between the local maxima in moment arm oscillations. After 48 h, the following examination was at 64 h; then, the fatigue test was run until the oscillation increase of the momentum arm exceeded 20%, which occurred at 112 h. As can be seen, such an experiment does not result in signal values that could be considered as directly preceding crossing the limits of the permissible material fatigue. Interestingly, the run-out decreases with an increase in oscillations of the moment arm up to 32%, much greater than the allowable 20%. A similar situation occurred in a previous examination where the increase in momentum arm oscillations above 20% occurred together with a local decrease of run-out values.

**Figure 14:**
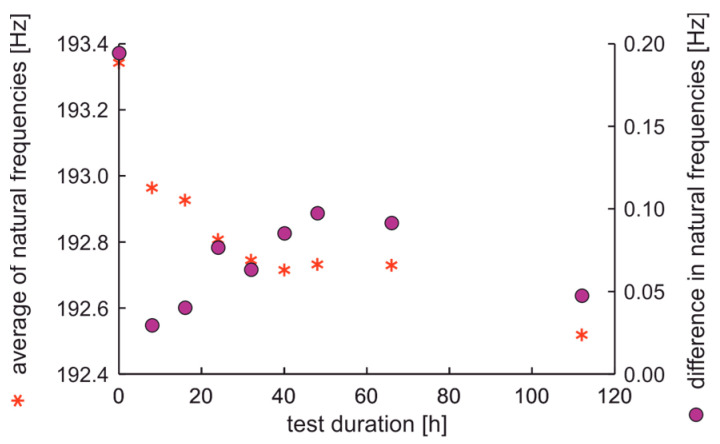
Average values and differences in natural frequencies of the new rim from ARM in an extended fatigue test.

The average values and differences in natural frequencies are presented in Figure 14. In this case, the average values and differences in natural frequencies initially decrease; this can be again related to mechanical running-in of the rim in initially new factory conditions. Higher values of differences in natural frequencies than 0.075 Hz are linked with a rim that does not meet run-out standards, see Figure 13.

**Figure 15:**
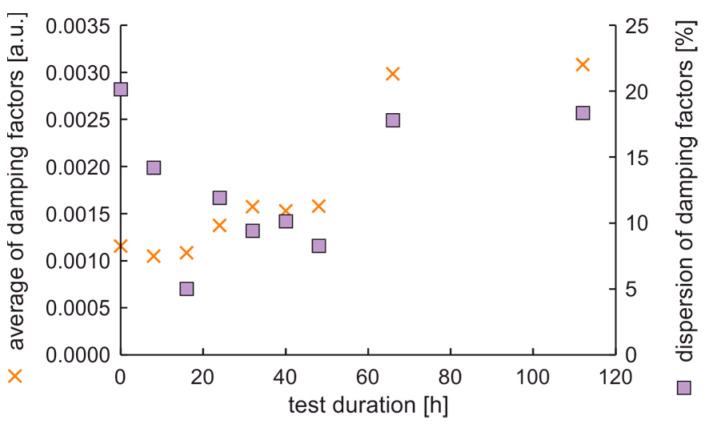
Average values and dispersion of damping factors of the new rim from ARM in an extended fatigue test.

The average values and dispersion of damping factors are presented in Figure 15. The results show that the average damping factor increases with the time of fatigue test duration. The dispersion of damping factor initially decreases when the test duration increases up to 16 h, then stabilizes and increases. The dispersion of damping factors versus test duration is not a monotonic function. The same as in the previous study, the maximum value of the dispersion of damping factors is related to the maximum increase of oscillations of the moment arm.

### 6.3. New Rim Type 6Jx15H2 of ARM, with a Rusty Hub—Examinations with the Use of the Fatigue Station

**Figure 16:**
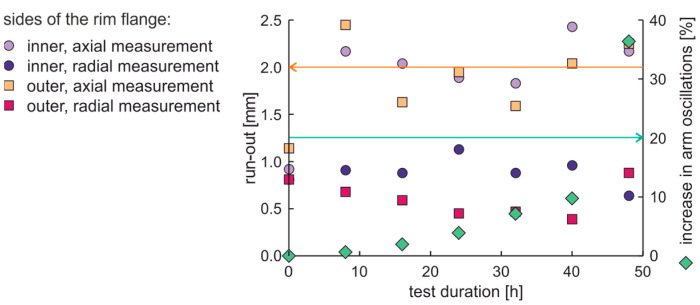
The axial and radial run-out and increase in moment arm oscillation values of the new rim from ARM in the fatigue test with a rusty hub.

The fatigue station for the dynamic wheel cornering fatigue test can also be used to simulate different rim–hub contacts. The current trial used a slightly rusty hub to mount the rim. The rim of the same ARM is examined as in Section 6.2. The axial and radial run-out values of the rim on the inner and outer sides of the rim flange are presented in Figure 16. Values indicate that the run-out norm was exceeded much earlier than in Section 6.1 and Section 6.2.

**Figure 17:**
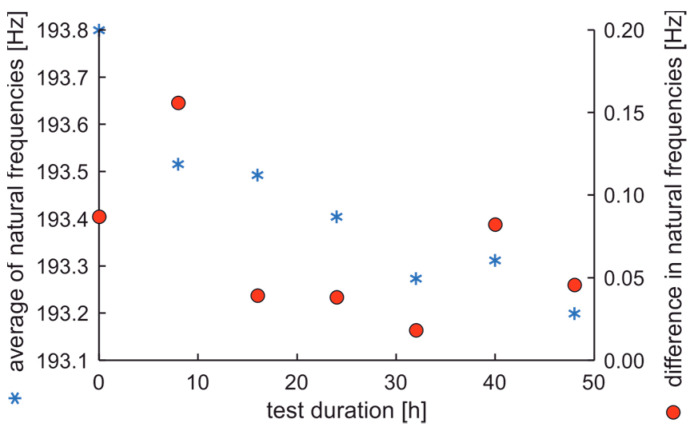
Average values and differences in natural frequencies of the new rim from ARM in the fatigue test with a rusty hub.

The increase in oscillation of the moment arm occurs at a time during the fatigue test similar to the test described in Section 6.2. For the present test, it occurs after 48 h, whereas previously, it occurred after 40 h and after 32 h. The average values and differences in natural frequencies are presented in Figure 17.

The course of the average natural frequency shows, as before (see Figure 10), a characteristic decrease during the fatigue test. However, the course of difference in natural frequencies is now characterized by greater values than presented in Figure 13, but it cannot be related to mechanical running-in of the rim in the initially new factory condition. The difference in natural frequencies is greater than 0.16 Hz at 8 h. This fact corresponds with the substantial run-out in the outer flange of the rim, whose axial deviation is 2.5 mm.

**Figure 18:**
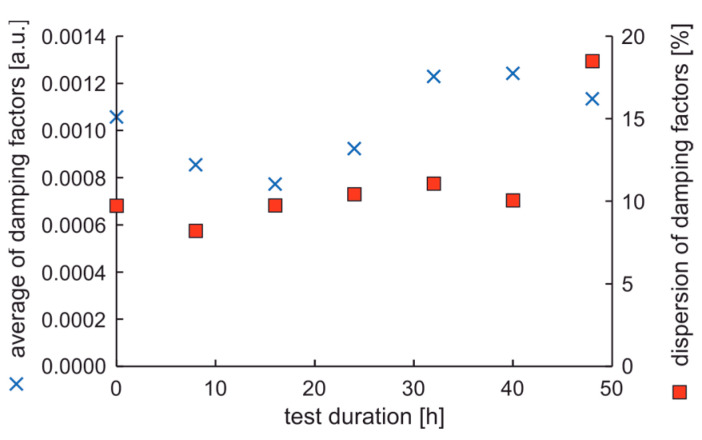
Average values and dispersion of damping factors of the new rim from ARM in the fatigue test with a rusty hub.

The average values and dispersion of damping factors are presented in Figure 18.

Again, the results show that the average of the damping factor initially decreases and then increases. Also, in this case, the dispersion of damping factors is highest when the moment arm’s oscillations increase is maximal. Also, the maximum dispersion of the damping factor is preceded by an increase in the average value of damping factors.

**Figure 19:**
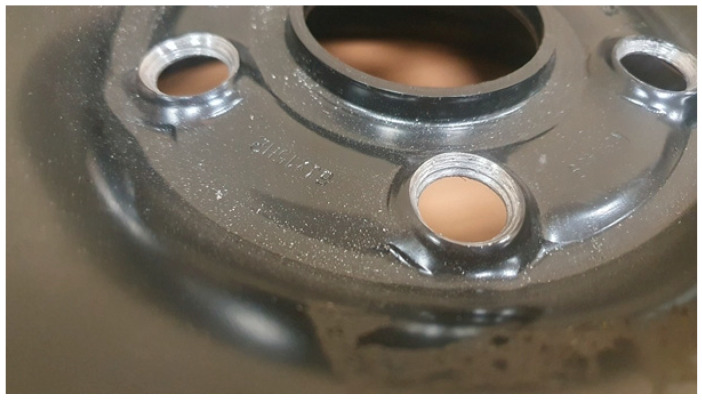
Degradation of bolt holes after 40 h of the fatigue test with rusty hub use for the new rim from ARM.

From the 40 h of test time, signs of degradation of the rim bolt holes and acoustic clicking were observed. The degradation of the rim bolt holes after 40 h of test duration is presented in Figure 19.

### 6.4. New Rims Type 6Jx15H2 of LCM

**Figure 20:**
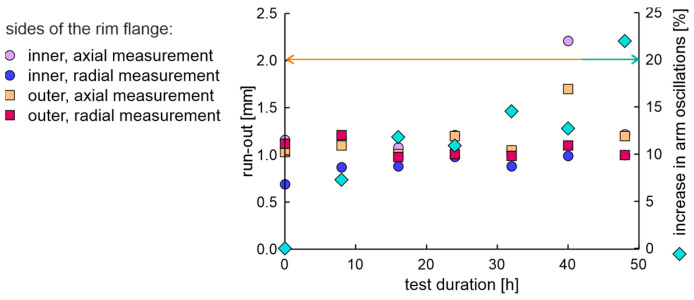
The axial and radial run-out values of the first new rim of LCM in a fatigue test.

New rims of LCM sometimes seem to be a good selection from an economic point of view. Thus, two new rims were used in examinations. The axial and radial run-out values of the rim on the inner and outer sides of the flange are presented in Figure 20. During the 40 h of the fatigue test, the PN standard is completely met, while the ETRTO standard is exceeded only for measurements after 8 and 24 h. These results are better than for the new rim of ARM.

**Figure 21:**
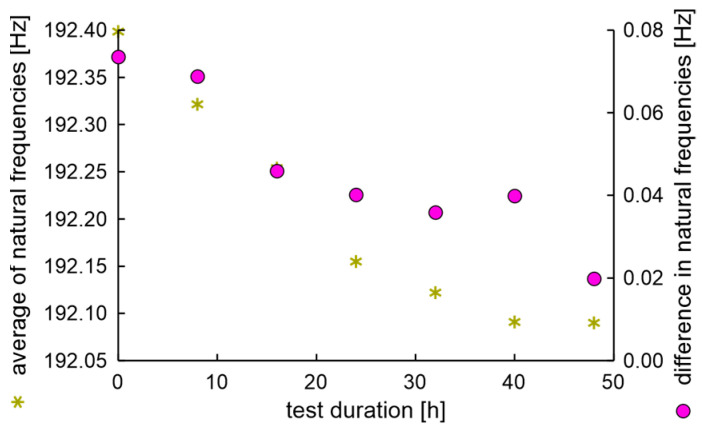
Average values and differences in natural frequencies of the first new rim of LCM.

The average values and differences in natural frequencies of the first rim of LCM are presented in Figure 21. The average of natural frequencies and the difference in natural frequencies’ tendency to decrease with test duration is evident.

**Figure 22:**
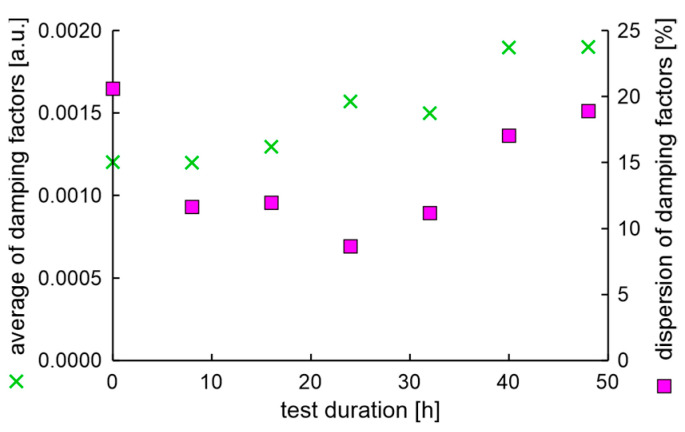
Average values and dispersion of damping factors of the first new rim of LCM in a fatigue test.

The average values and dispersion of damping factors of the first rim of LCM are presented in Figure 22. The dispersion of damping factors’ maximum is again at the end of the test and corresponds with the time of maximum increase of moment arm oscillations. The only issue that can be found in the first new rim of LCM is the high initial value of the dispersion of damping factors. However, this value unexpectedly decreases during the fatigue test.

**Figure 23:**
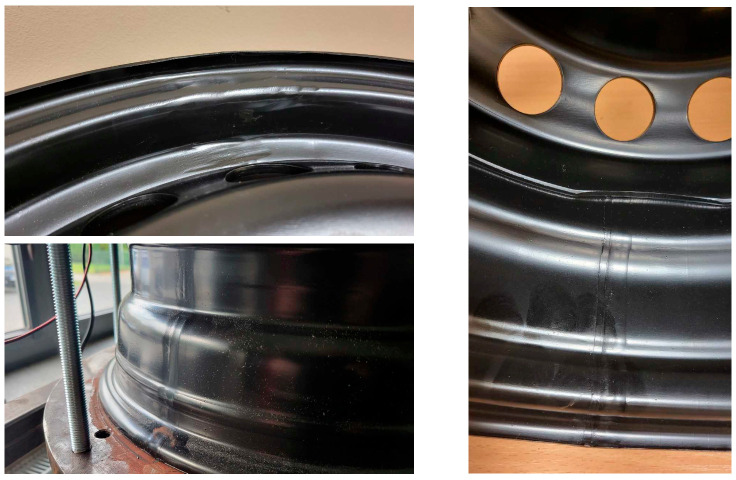
Views of imperfections of the second rim from LCM.

The second rim of LCM was not so fine. Its run-out values do not fit with the ETRTO and PN standards; its construction imperfections are visible in Figure 23. For reasons of safety of the testing personnel, this wheel was not subjected to fatigue tests.

### 6.5. Used Rim from OEM That Meets the ETRTO Standard but Does Not Meet the PN-93S Standard

**Figure 24:**
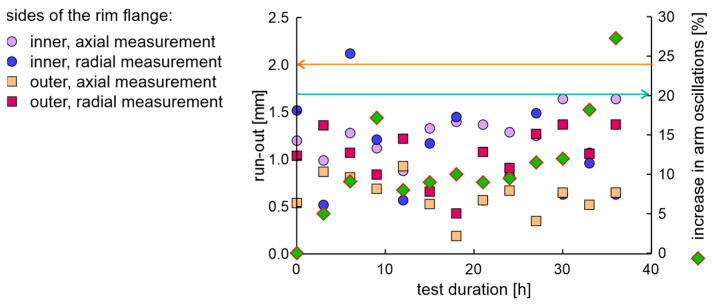
The increase of moment arm oscillations and run-out values of the used rim of OEM in a fatigue test.

The used rim from OEM with an initial condition meeting the ETRTO standard but not meeting the PN-93S standard was examined. Based on visual inspection and run-out measurements, it can be intuitively concluded that the tested rim is minimally worn. However, as the exact wear characteristics of the rim before the tests were not known, the fatigue cycle interval was reduced from 8 to 3 h. The increase of moment arm oscillations and axial and radial run-out values of the rim on the inner and outer sides of the flange are presented in Figure 24.

**Figure 25:**
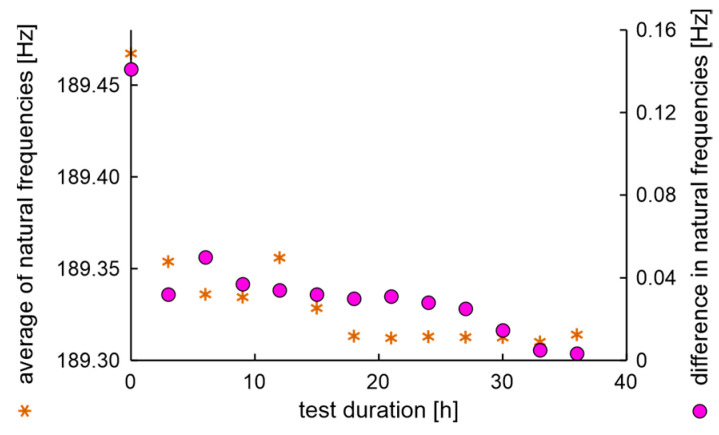
Average values and differences in natural frequencies of the used rim of OEM in a standard fatigue test with reduced time between EMA tests.

The obtained results of the run-out indicate that after 6 h of the fatigue test, the rim lost its dimensional tolerance, but its shape somehow regenerated. It can be noticed that the used rim lost its dimensional tolerance much earlier than a new rim from the same OEM (Figure 10 Section 6.1). The average values and differences in natural frequencies are presented in Figure 25.

**Figure 26:**
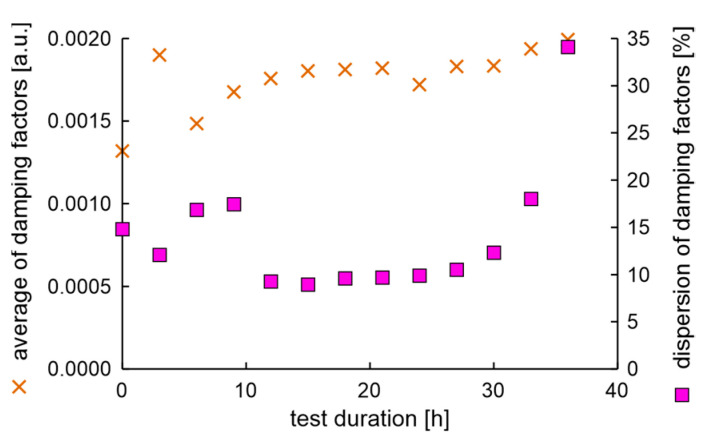
Average values and dispersion of damping factors of the used rim of OEM in the fatigue test.

The EMA initial test results show that the difference in natural frequencies is more significant than that obtained for the new rim of the same producer, which can be expected, as the rim was used. Interestingly, the local maximum difference in natural frequencies at 6 h correlates with the maximum run-out. The average values and dispersion of damping factors are presented in Figure 26.

As in the previous examination, the average of damping factor values increased in a non-monotonic way, while the dispersion of damping factors exceeded 17% sometime after the dimension degradation point. Also, the maximum dispersion of damping factors is correlated with the maximum increase of moment arm oscillations.

### 6.6. New Rims Type 6Jx15H2 of OEM—Examination of the Rim with Artificially Generated Cracks

**Figure 27:**
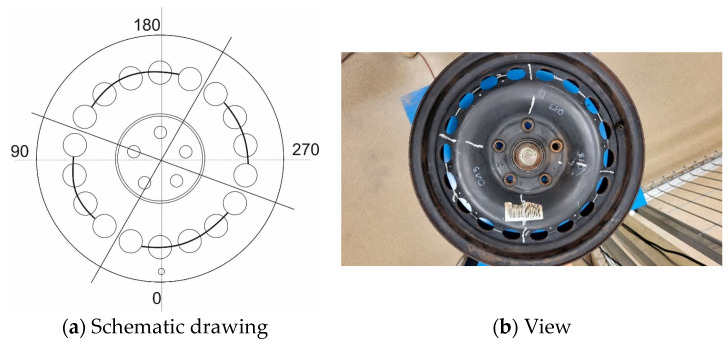
Artificially formed cracks on the rim disc: (**a**) Schematic drawing; (**b**) View.

One of the typical places of rim cracks is located between the vent holes. Waiting for such rim degradation to be produced by the fatigue station is problematic from the experiment’s security point of view. However, artificially made cracks of the rim enable a controlled investigation of parameters of the degraded rim. The artificial cracks were realized by mechanical cutting along the red lines marked in Figure 27a. The effects are shown in Figure 27b.

**Figure 28:**
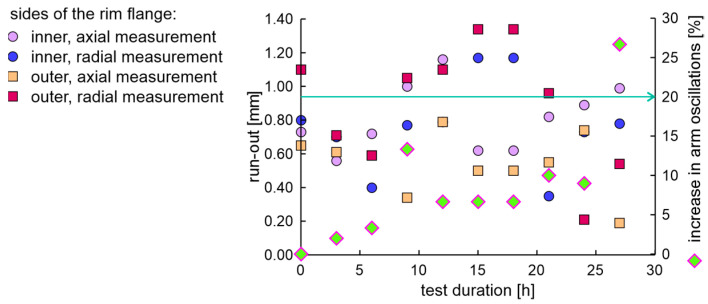
The increase in moment arm oscillations and run-out values of the new cut rim from OEM in a fatigue test.

The cuttings resulted in a 4 Hz decrease in natural frequency while the damping factor remained constant. Due to rim design modifications, the fatigue cycle interval was reduced from 8 to 3 h. Axial and radial run-out values of the cut rim presented in Figure 28 are not subject to serious rim quality suspicions.

**Figure 29:**
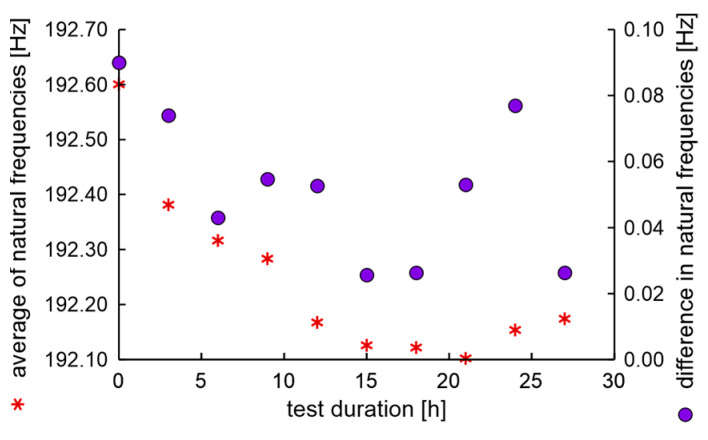
Average values and differences in natural frequencies of the cut rim of OEM in a standard fatigue test.

The end condition of the test occurred in 27 h, which is faster than for any other new, undamaged rim. However, the run-out values, measured during the fatigue test of the rim with artificially generated cracks, are much smaller than for unmodified rims. The characteristics of natural frequencies during the fatigue test presented in Figure 29 appear normal.

**Figure 30:**
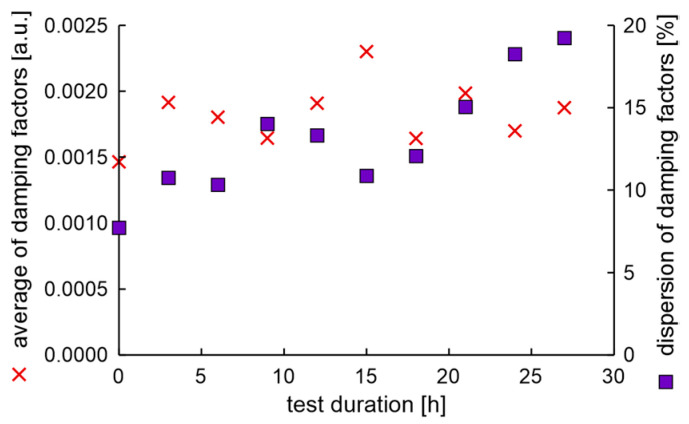
Average values and dispersion of damping factors of the cut rim of OEM in a fatigue test.

Measured damping factors during fatigue experiments presented in Figure 30 are also similar to the results obtained in previous sections.

Again, most importantly, the maximum dispersion of the damping factor happens at the same time as the maximum increase of moment arm oscillations.

## 7. Discussion of Results of Section 6

The discussion considers cyclic destructive fatigue tests separated by non-destructive classical visual inspection, rim run-out tests, and the application of experimental modal tests presented in Section 6, as well as the verification results included in this section. The first result of the comparative studies presented in Section 6.1 and Section 6.2 is that the cyclic use of the fatigue station and the coupled wheel rim tests give much more information than the continuous use of the fatigue station until the fatigue test end condition is reached.

The results of the cyclic investigations indicate several repetitive relationships. The EMA shows that a downward trend characterizes the course of the natural frequency versus fatigue test duration. However, regardless of which rim is tested, the first period of the fatigue test causes the greatest decrease in frequency. Thus, the initial reduction in natural frequencies can be interpreted as the fatigue station performing a peculiar mechanical process on the tested rim. Moreover, the natural frequencies of rims with cracks can differ significantly, for example, by 4 Hz (in Section 6.6–192.64 Hz). However, the direct comparison of the real initial natural frequency of rims can be deceptive; for example, a homogeneous rim from OEM natural frequency was 192.56 Hz (Section 6.1), while a cracked rim from OEM was 192.64 Hz. Therefore, an analysis of the technical condition of the wheel based on natural frequencies is only possible if its exact historical data is known.

Moreover, looking at the results of the measurement of natural frequency differences determined by Formula (1) in Section 4 and relating them to the examination in the low band of the new rim (Figure 7), one can point out that, in the proposed experimental set-up, the result of natural frequencies mixing is masked by the constant component of experimental FFT. The FFT width is about 0.4 Hz, while the difference in natural frequencies is lower than 0.2 Hz. However, based on the simulation and the results described in Section 7.2, geometric damage to the rims greater than 2.5 mm should produce an unmasked signal in a low band of mixing resonant modes. Therefore, the precise analysis of the technical condition of the wheel based on the low band requires a more advanced measurement system than the one presented in this publication.

Separate analysis of the rim run-out measurements obtained during the fatigue test can lead to the conclusion that determining the technical condition of the wheel rim according to run-out standards does not always make sense. The run-out values of the rim during the experiment are usually variable. Rim run-out values typically initially meet the standard, then may not meet it, or may be reduced and meet the standard again. It can be seen in almost all experiments, for example, in Section 6.5 in Figure 24 and Section 6.4 in Figure 20. In contrast, the averages of damping factors and dispersion of damping factors versus fatigue test duration are characterized by an upward trend.

### 7.1. Precise Technical Conditions Determination of Wheel Rims with the Use of Data Fusion

The real task is to determine the technical condition of a wheel of unknown history and any type using non-destructive testing. For this purpose, the data fusion of different methods can be helpful. The first and most important relation to be observed in the presented experiments is that the maximum increase in moment arm oscillations is related to the maximum dispersion of damping factors. Such data fusion is shown in Table 3.

The first result of data fusion is that the minimum dispersion of damping factors, corresponding to an unacceptable increase in moment arm oscillations, is 18.4%. To threshold detection, the maximum value of the dispersion of damping factors that corresponds to an acceptable increase in moment arm oscillations is required. This data fusion of the cycle before the maximum increase in moment arm oscillations with the dispersion of damping factors is presented in Table 4. The maximum acceptable value of the dispersion of damping factors is 18.0%.

As the dispersion of damping factors is determined with 0.1% tolerance, the threshold between acceptable and not acceptable technical conditions of the rim can be set at 18.2%.

### 7.2. Field Testing—Used Rims Type 51/2Jx14H2 of OEM

**Figure 31:**
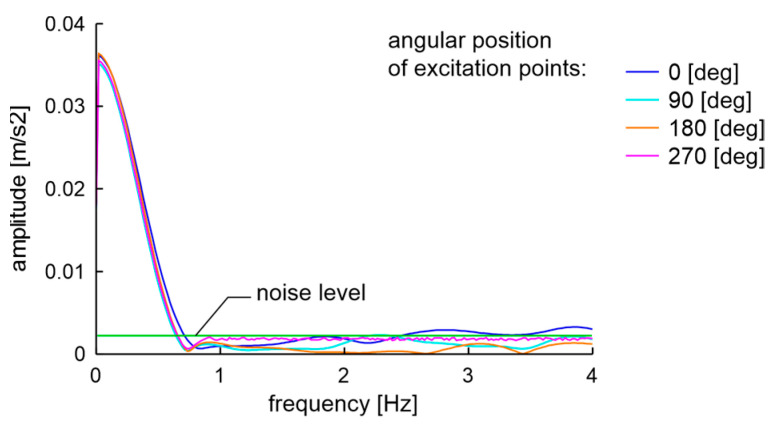
Low band spectra of EMA of 51/2Jx14H2 rim in an acceptable technical condition with a visual and run-out examination at the beginning of the experiment.

The actual task is to determine the technical condition of a wheel of unknown history and any type using non-destructive testing. The examinations of the used 14-inch rim of type 51/2Jx14H2 from OEM are presented here. One rim was used, and the other was brand new. The initial state of those rims described with the run-out standards meets the ETRTO standard but does not meet the PN-93S standard. The used rim parameters are presented in Table 5. The initial visual condition of the used rim can be described as acceptable, as there is no visible rust or cracks, and the air does not leak from the tire. In such a state, the low band vibration excited of the rim is presented in Figure 31.

However, the visual and run-out examination does not involve EMA and the value of dispersion of damping factors, which in this case is 22.4%. Thus, according to the presented investigation, this rim must be classified as unfit for use. However, an experimental verification of this statement must be conducted. As experimental conditions, classic cases of mechanical damage to the rim were assumed. Hitting the curb of a nominal load vehicle with adequately inflated wheels and operation on partially paved forest roads with numerous uplifts caused by the roots of old trees were tested. For safety reasons, as the used rim was classified as unfit, this experiment was performed for a small trailer towed by a passenger car with one used and one new rim.

**Figure 32:**
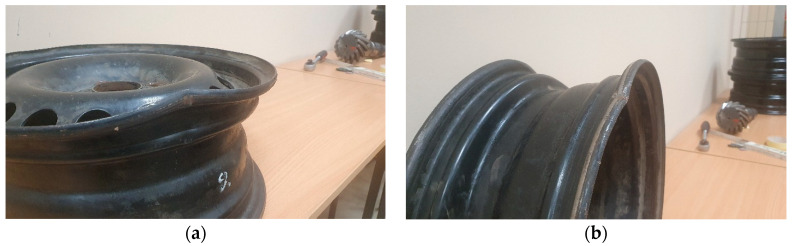
View of rim: (**a**) After it hit a curb; (**b**) Further six months of operation on partially paved forest roads.

The first examination was hitting the curb at 20 km/h. The second examination was a six-month operation on partially paved forest roads with numerous uplifts caused by the roots of old trees. During the first experiment, the damage of the used rim was significant, as shown in the run-out values and the view presented in Figure 32, while nothing occurred to the new rim.

The run-out values of the used rim during the experiment are presented in Table 5. It is visible that the rim does not meet the run-out standards after hitting the curb. However, interestingly, the wheel passed the soapy water test for rim–tire integrity. Therefore, further operation was on non-public roads.

**Figure 33:**
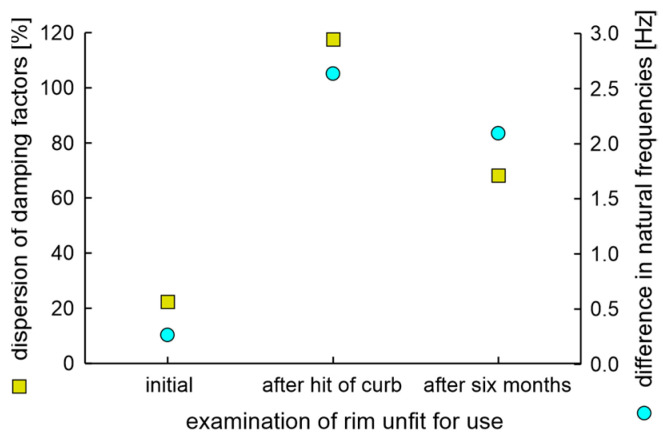
Differences in natural frequencies and dispersion of damping factors of unfit for use rim type 51/2Jx14H2 of OEM.

The obtained results show that during operation in the conditions of forest roads, the heavy degradation of the radial component of the geometrical dimension somehow decreases. Differences in natural frequencies and the dispersion of damping factors of the used rim are presented in Figure 33, which confirms this thesis. Still, all measured values here are much higher than in previously submitted laboratory cases of rim degradation with fatigue station use, as shown in Section 6.1, Section 6.2, Section 6.3, Section 6.4 and Section 6.5.

**Figure 34:**
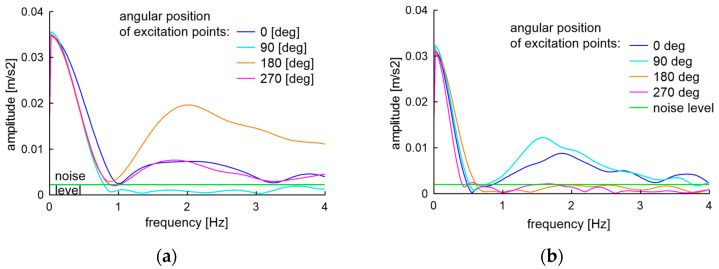
Low band signals of unfit for use rim: (**a**) After a hit of the curb; (**b**) Further six months of operation on partially paved forest roads.

The low band signals after two experiments of unfit for use rims are presented in Figure 34. Characteristics shown in Figure 34 confirm the assumptions of the measured difference in natural frequencies. The result of frequency mixing is visible in the low band when differences in natural frequencies are greater than 0.4 Hz. The soapy water test carried out at the end of the sixth month of operation showed a loss of integrity of the unfit for use rim and tire and the examination was finished.

The postulate that the technical condition of the rim is related to the dispersion of damping factors, and the possibility of classification of technical condition based on simple threshold criteria has been proved. The 14-inch wheel with 22.4% dispersion of damping factors was unacceptably deformed when driving over a curb at 20 km/h. The rim with the initial dispersion of damping factor 8.1 passed all driving tests, including hitting the curb of a nominal load vehicle with properly inflated wheels and operation on partially paved forest roads.

### 7.3. Prediction of Remaining Time of Use

The prediction of the remaining service life of a wheel rim in relation to its unknown mileage and the unknown nature of its operation must be based on the determination of its current technical condition and signals prior to its unusable state. In Section 7.1, we have shown that the current technical condition of a wheel rim can be precisely determined by the threshold value of the damping factors dispersion. As a signal prior to the unusable state, a characteristic signal with repeating features should be selected. In almost all cases, the time course of the moment arm oscillation is similar to the shape of the μ mark. The first local maximum is lower than the second. The second maximum is in our examination always the termination of the fatigue test; for example, see Figure 10. The only exception was in Section 6.3 in the case of a rusty hub. However, the use of oscillations of moment arm to define or predict the remaining time of use is at least a semi-destructive test. Therefore, it is essential to check if other parameters measured in non-destructive tests can indicate that the threshold value mentioned is approaching, by way of the time relation analysis from the experiment termination. The time relation refers to a set of research sequences preceding the end of the experiment:

(3) $$\{T\left(te-l\right)\},$$

where *T* is the time before the end of the experiment (*te*) preceded by the number of sequences equal to *l*. The cyclic change of characteristic parameters can be described with the fold change *FC*(*P*) according to Equation (4):

(4) $$FC(P)=\frac{P(T\left(n\right))}{P(T(m)},$$

where *P* is the measured parameter from the set {RO—run-out, *aDF*—an average of damping factors, *dDF*—dispersion of damping factors}, *n* and *m* are numbers of examination sequence where *n* < *m*. The above definitions allow for the construction of Table 6, in which the parameters indicating that the threshold value of the dissipation of damping factors is approaching are characterized.

The data in Table 6 show that there is a set of parameters that indicate the approaching end of service life. In three out of five cases, the end of the rim’s service life is preceded by a fold change in the dispersion of the damping factor greater or equal to 1.46. It seems that this parameter fold change can act as a separate condition of indication. In four out of five cases, the end of the rim service life is preceded by a fold change in the average of damping factors. In three cases out of four, the fold change of the average of damping factors is correlated with an increased value of run-out greater than 2.2 mm. The fold change in the average of damping factors is coupled with the fold change of dispersion of damping factors for the experiment depicted in Section 6.2, which was conducted with different sequences. Thus, the binary indicator of approaching the end of the steel car wheel rim can be written in the form of Formula (5):

(5) $$I=\left\{\begin{array}{cc} 1 & FC\left(dDF\right)>1.71\\ 1 & FC\left(aDF\right)>1.33\;and\;RO>2.43\\ 0 & in\;other\;cases \end{array}\right\},\;$$

where *I* is the binary indicator, while the other designations are as in Equation (4). Unfortunately, calculating the proposed indicator requires at least two consecutive rim tests for the vehicle’s standard annual mileage. According to our estimations implemented here, when the indicator equals one, the expected left mileage in mixed traffic conditions is about 9600 km. When the indicator is equal to zero, the rim can be used without fear.

### 7.4. Presented Method Versus Computer Vision Methods

It should be noted that the presented method uses data fusion of modal analysis and geometrical measurements to identify the technical condition and indicate the approaching end of the service life of the steel automotive wheel rims. This method belongs to the noninvasive measurement group. However, the noninvasive measurement group is wider. Computer vision techniques are also used to analyze automotive wheel rims; see Table 7.

The detection results of the proposed method shown in Section 6 and the computer vision methods are quite different. The methods presented in Table 7 are primarily oriented to be implemented at the rim factories, while the proposed method is intended to be used at car repair stations.

## 8. Conclusions

A set of conclusions can be grouped depending on the type of investigation methods: simulation, laboratory experiment, field trial case examinations, and data fusion.

The simulation results of modal analysis enable the proper construction of laboratory measurement systems and fatigue stations. Analysis of traffic conditions, service maintenance, and standards applied by rim producers of steel car wheel rims enable the proposition of cyclic examination, including initial measurement and following fatigue and measurement sequences. The presented investigation shows that sequential fatigue and rim characterization provide many more possibilities for the analysis of the character of wheel rim parameters than standard continuous tests until the end of fatigue test condition is met.

The practical side of the numerical modal analysis shows the possibility to define the degree of the rim degradation based on the variability of natural frequencies, whereby when the scale of the damage is greater, the bigger the frequency difference. According to simulations, the difference in natural frequencies for an ideal automotive rim should be lower than 0.21 Hz, while the difference may be as high as 12 Hz for a completely damaged rim. Experiments confirm that values of peaks for an ideal rim that met standards are below 0.2 Hz and for significantly degraded rim, the differences can reach 4 Hz. Experimental modal analysis also shows that the difference of natural frequencies is related to low band signals which range from 0 to 4 Hz, as these signals come from excited frequencies mixing. Thus, peaks at the low band can be a measure of geometrical rim degradation.

The experimental data shows that processes of wheel rim degradation are too complex to describe the technical condition of the rim with a single standard or one basic parameter. Our examination shows that the dispersion of damping factors of vibration excited at four points that are spread angular at the outer rim flange can act as a precise tool for binary identification of the technical condition of the rim with an unknown history of exploitation. The dispersion of damping factors acts as the threshold value for acceptance of the material health of the rim. We showed that this parameter is strongly related to the increase of momentum arm oscillations depicted in standards of precise rim factory testing with the destructive method. At the same time, the dispersion of damping factors can be measured in a non-destructive way, and it appears to work for rims with an unknown history. Moreover, the threshold value obtained in experiments for a 15-inch rim works appropriately for a 14-inch rim.

A more complex situation is related to predicting the end of the service life of the rim. This prediction may be based on natural frequencies, damping factors, and manufacturer data. However, that is only possible if its exact sequence of historical data, e.g., collected by the dealership during routine inspections, is known, and if the vehicle is in ideal condition and exploited in standard conditions. However, the technical condition of a vehicle can affect the expected remaining time of rim use.

On the other hand, the proposed examination results give an indication of the approaching end of rim service life with acceptable complexity. The indication requires two sequential examinations of the rim in standard periods related to the average annual mileage of the vehicle. For the proposed method, the data must include two sets of three parameters: run-out, average of damping factors, and dispersion of damping factors. On this basis, the indication of approaching the end of rim service life is advisable in advance, which can be described as about 10,000 km left of mileage.

The current limitation of the presented method is that it was tested only for steel car wheel rims. Given the above, the natural direction of further work is to show the technique’s operation for automotive alloy rims and rims for particular purposes, e.g., for agricultural and military vehicles. Of course, expanding the area of research will force the automation of data processing and access. Furthermore, increases in data will enable statistical analysis of the methodology. However, this research will require a significant investment in time.

## Figures and Tables

**Table 1:** Rim types under examination with initial run-out characterization.

Rim Manufacturer	Rim Type	Rim Category	ETRTO	PN-93S	Run-Out on Flange < 2 mm	Examination in Section-Type of Study
OEM	6Jx15H2	new	met	not met	met	6.1—sequential
ARM	6Jx15H2	new	not met	not met	met	6.2—sequential and then continuous
ARM	6Jx15H2	new	met	met	met	6.3—rusted hub
LCM *	6Jx15H2	new	met	met	met	6.4—sequential
OEM	6Jx15H2	used	met	not met	met	6.5—sequential
OEM	6Jx15H2	new	met	met	met	6.6—artificial fatigue cracks
OEM	51/2Jx14H2	used	met	not met	met	7.2—field testing

* The second rim of this manufacturer, despite being new, was not in acceptable technical condition to be tested as the run-out on the outer flange was over 2.2 mm and because of other issues.

**Table 2:** Summary of results presented in Figure 2, Figure 3 and Figure 4.

Case	Mode Number	Maximum Amplitude of Vibration [mm]	Variability of Maximum Amplitudes [mm]	Natural Frequency [Hz]	Variability of Natural Frequencies [Hz]
Ideal rim	3	0.8353	0.0005	209.35	0.21
	4	0.8358		209.56	
Inner hole in the barrel	3	0.8339	0.0050	209.98	0.40
	4	0.8289		210,58	
Center hole in the barrel	3	0.8329	0.0018	209.29	0.51
	4	0.8311		209.80	
Outer hole in the barrel	3	0.8347	0.0019	208.96	0.48
	4	0.8366		209.48	
Broken part of the flange	3	1.1140	0.2818	197.19	11.99
	4	0.8322		209.18	

**Table 3:** Data fusion of maximum increase in moment arm oscillations and maximum of dispersion of damping factors.

Examination Section	Time of Test [h]	Maximum of Increase in Moment Arm Oscillations [%]	Maximum of Dispersion of Damping Factors [%]
6.1	40	23.2	26.2
6.2	112	31.5	18.4
6.3	48	36.3	18.5
6.4	48	22.0	18.9
6.5	36	27.3	34.1

**Table 4:** Data fusion of cycle before maximum increase in moment arm oscillations with the dispersion of damping factors.

Examination Section	Time of Test [h]	Maximum of Increase in Moment Arm Oscillations [%]	Maximum of Dispersion of Damping Factors [%]
6.1	32	11.1	11.2
6.2	32	20.0 *	9.4
	66	10.7	17.8
6.3	40	9.7	10.1
6.4	40	12.7	17.1
6.5	33	18.2	18.0

* Local maximum of increase in moment arm oscillations.

**Table 5:** Run-out values of the rim during the fatigue experiment in natural heavy conditions.

Examination	Run-Out [mm]					
	Inner Flange		Outer Flange		Standard	
	Axial	Radial	Axial	Radial	PN-93S	ETRTO
Initial (used rim)	1.65	1.02	0.64	0.57	not met	met
After hitting the curb	4.35	2.75	4.52	2.5	not met	not met
After six months of operation on forest roads	3.53	1.77	2.92	1.12	not met	not met

**Table 6:** The set of parameters indicating that the threshold value of the dissipation of damping factors is approaching.

Examination Section	Characteristic Sign before Maximum Dispersion of Damping Factors	The Characteristic Sign and Its Value	Time Relation	Time Indicated [h]
6.1	run-out of inner side of flange > 2.2 mm	2.71 mm	{*T*(*te*−1)}	8
	local increase in the average of damping factors	*FC*(*aDF*) = 1.76	{*T*(*te*−2), *T*(*te*−1)}	8
6.2	local increase in the average of damping factors	*FC*(*aDF*) = 1.89	{*T*(*te*−2), *T*(*te*−1)}	46 *
	local increase of dispersion of damping factors	*FC*(*dDF*) = 2.15	{*T*(*te*−2), *T*(*te*−1)}	46 *
6.3	run-out of inner side of flange > 2.2 mm	2.43 mm	{*T*(*te*−1)}	8
	local increase in average of damping factors	*FC*(*aDF*) = 1.33	{*T*(*te*−3), *T*(*te*−2)}	16
6.4	run-out of inner side of flange > 2.2 mm	2.21 mm	{*T*(*te*−1)}	8
	local increase in average of damping factors	*FC*(*aDF*) = 1.26	{*T*(*te*−2), *T*(*te*−1)}	8
	local increase of dispersion of damping factors	*FC*(*dDF*) = 1.53	{*T*(*te*−2), *T*(*te*−1)}	8
6.5	local increase of dispersion of damping factors	*FC*(*dDF*) = 1.46	{*T*(*te*−2), *T*(*te*−1)}	3
		*FC*(*dDF*) = 1.71	{*T*(*te*−3), *T*(*te*−1)}	6

* With the aging procedure in accordance with the standard, no aging sequence with a time equal to 8 h before the end of the experiment.

**Table 7:** Sample of noninvasive automotive wheel rim examinations based on computer vision.

Method	Requirements	Method Application	Examined Object	Detection Result	Ref.
X-ray, digital radiography (DR)	radioscopic images taken at different positions and reference image	image segmentation analysis	aluminum castings	internal cracks in the material structure	[67]
X-ray, digital radiography (DR)	series of images with different radiation intensity	adaptive threshold and morphological reconstruction of structure	automotive aluminum rim	internal defects such as gas holes and shrinkage cavities	[68]
X-ray, NIR photography	radioscopic and NIR images of the same object	convolutional neural network application to image recognition	automotive aluminum rim	internal casting defects	[69]
VIS photography	series of photography of multiple views	feature analysis in numerous views	automotive aluminum rim	geometry imperfections	[70]

## Data Availability

Data are contained within the article.
